# Targeting of adipose tissue macrophages by bee venom phospholipase A2 attenuates high-fat diet-induced obesity

**DOI:** 10.1038/s41366-021-00823-4

**Published:** 2021-05-04

**Authors:** Hyunju Jeong, Chanju Lee, Chenyu Cheng, Hung Chun Chou, HyeJin Yang, Hyunsu Bae

**Affiliations:** Department of Physiology, College of Korean Medicine, Kyung Hee University, Seoul, Republic of Korea

**Keywords:** Inflammatory diseases, Obesity

## Abstract

**Background/objectives:**

Adipose tissue macrophages (ATMs) exist in either the M1 or M2 form. The anti-inflammatory M2 ATMs accumulate in lean individuals, whereas the pro-inflammatory M1 ATMs accumulate in obese individuals. Bee venom phospholipase A2 (bvPLA2), a major component in honeybee (Apis mellifera) venom, exerts potent anti-inflammatory effects via interactions with regulatory T cells (Treg) and macrophages. This study investigated the effects of bvPLA2 on a high-fat diet (HFD)-induced obesity in mice.

**Subjects/methods:**

For in vivo experiments, male C57BL/6, CD206-deficient, and Treg-depleted mice models were fed either a normal diet 41.86 kJ (ND, 10 kcal% fat) or high-fat diet 251.16 kJ (HFD, 60 kcal% fat). Each group was i.p. injected with PBS or bvPLA2 (0.5 mg/kg) every 3 days for 11 weeks. Body weight and food intake were measured weekly. Histological changes in the white adipose tissue (WAT), liver, and kidney as well as the immune phenotypes of the WAT were examined. Immune cells, cytokines, and lipid profiles were also evaluated. The direct effects of bvPLA2 on 3T3-L1 pre-adipocytes and bone marrow-derived macrophages were measured in vitro.

**Results:**

bvPLA2 markedly decreased bodyweight in HFD-fed mice. bvPLA2 treatment also decreased lipid accumulation in the liver and reduced kidney inflammation in the mice. It was confirmed that bvPLA2 exerted immunomodulatory effects through the CD206 receptor. In addition, bvPLA2 decreased M1 ATM and alleviated the M1/M2 imbalance in vivo. However, bvPLA2 did not directly inhibit adipogenesis in the 3T3-L1 adipose cells in vitro.

**Conclusions:**

bvPLA2 is a potential therapeutic strategy for the management of obesity by regulating adipose tissue macrophage homeostasis.

## Introduction

Obesity is a growing global health problem that is characterized by the excess accumulation of body fat and a high body mass index (BMI ≥ 30) [1, 2]. This global epidemic has been identified as a major health risk that reduces the quality of life and increases the mortality rate due to insulin resistance. Obesity is often accompanied by other diseases, such as type 2 diabetes, cardiovascular disease, hypertension, cancer, polycystic osteoarthritis, and metabolic disorder. Treatment of obesity requires long-term, lifestyle management (healthy eating and exercise); however, its effects can be extremely difficult to control. The development of novel treatments, without adverse effects, remains a priority in healthcare [3–5].

Excessive accumulation of fat in white adipose tissue (WAT) triggers the improper release of adipokines, free fatty acids (FFAs), and triglycerides (TG) from adipocytes and accelerates the activation of IκB kinase (IKK), c-Jun N-terminal kinase, and the Nuclear factor kappa B pathways, resulting in insulin resistance and chronic inflammation [6, 7]. Furthermore, it has been reported that the accumulation of immune cells, such as macrophages, monocytes, and lymphocytes, in adipose tissue (AT) is increased in the obese state. It has been shown that most of these immune cells infiltrating the AT are macrophages [8, 9]. Adipose tissue macrophages (ATMs) have been known to rapidly increase in the early stages of obesity, inducing inflammation and adipocyte apoptosis [10–12]. There are two types of ATMs, classically activated M1 macrophages and alternatively activated M2 macrophages [13]. M1 ATMs release pro-inflammatory cytokines, such as TNF-α, INF-γ, IL-1β, IL-6, and MCP-1. The recruitment of M1 ATMs in the AT leads to insulin resistance and adipocyte dysfunction [14]. In contrast, M2 ATMs secrete anti-inflammatory cytokines, such as IL-4, IL-10, and IL-13, and highly express Arg1 and Ym1 [15, 16]. In the lean state, most of the resident macrophages in the AT are polarized to the M2 phenotype, and they maintain normal adipocyte function via tissue remodeling and angiogenesis [17]. Previously, it was reported that macrophages express mixed M1/M2 forms in response to high-fat diet (HFD), but continued HFD induces more of the M2-like form of macrophages [18]. The M1/M2 imbalance toward the M1 phenotype results in inflammation and adipocyte dysfunction, whereas switching from the M1 to M2 phenotype improves the browning reaction of the WAT and insulin sensitivity [19]. Hence, targeting ATMs as a therapeutic strategy could be important for the treatment of obesity [20–22]. Phospholipase A2 (PLA2) is one of the major components in honeybee (*Apis mellifera*) venom. It has been shown that the secreted PLA2 isoform (sPLA2-V) and group VIA PLA2 (PLA2-VIA) are involved in polarizing macrophages to the M2 form in obesity [23, 24]. Distinct from sPLA2-V and iPLA2-VIA, bee venom PLA2 (bvPLA2) has also exhibited therapeutic effects on Parkinson’s disease, Alzheimer’s disease, pulmonary inflammation, and atopic dermatitis through an increase in the Foxp3+ regulatory T-cell populations (Treg) resulting in reduced inflammation [25–28]. Tregs play a major role in maintaining immune tolerance by suppressing effector T cells, B cells, and other myeloid lineage cells that induce macrophages. According to previous reports, bvPLA2 binds to dendritic cells via the CD206 mannose receptors. When bvPLA2 binds to the surface of dendritic cells, prostaglandin E2 (PGE2) secretion is increased via stimulation of cyclooxygenase-2 (COX-2), resulting in the differentiation of Foxp3^+^ regulatory T cells (Treg) via EP2 receptor signaling in Th0 cells [25]. However, the direct interaction of bvPLA2 on macrophages is still not well understood, despite the fact that macrophages abundantly express CD206. The proven various and strong immune modulatory effects of bvPLA2 supports its application in inflammation-associated obesity. In this study, we investigated the effect of bvPLA2 on macrophage inflammation in a HFD-induced obesity mouse model.

## Materials and methods

### Materials

Bee venom phospholipase A2 (bvPLA2, *Apis mellifera*), 3-Isobutyl-1-methylxanthine (IBMX), Dexamethasone (DEX), insulin, and oil red O were purchased from Sigma-Aldrich (St. Louis, MO, USA).

### Experimental animals

Male C57BL/6 mice (age, 5 weeks; weight, 18–20 g) and CD206^−/−^ mice (B6.129P2-*MRC1*^*tm1Mnz*^/J) were purchased from Jackson Laboratory (Bar Harbor, ME). After 1 week of adaptation (at 23 ± 2 °C, 60 ± 10% humidity, and a 12-h light/dark period), mice were fed either 41.86 kJ normal diet (ND; 10 kcal% fat, 20 kcal% protein, and 70 kcal% carbohydrate) or 251.16 kJ high-fat diet (HFD; 60 kcal% fat, 20 kcal% protein, and 20 kcal% carbohydrate) for 15 weeks (Research Diets, NJ, USA). The mice were intraperitoneally injected (i.p.) with PBS or bvPLA2 (0.5 mg/kg) every 3 days starting on week 5, and continuing for 11 weeks. For regulatory T cell (Treg) depletion, anti-mouse CD25 (clone: PC61) was produced in-house from hybridomas obtained from the American Type Culture Collection (ATCC; Manassas, VA, USA). The mice were i.p. injected with anti-mouse CD25 or rat IgG (Sigma-Aldrich) (0.5 mg/kg) every 3 days, for 11 weeks. The animal studies were approved by the University of Kyung Hee Institutional Animal Care and Use of Committee (KHUASP(SE)-19-004).

### Cell culture and differentiation

3T3-L1 pre-adipocytes were purchased from the Korean Cell Line Bank (KCLB, Seoul, Korea). The cells were cultured in DMEM (Welgene, Daegu, Korea) supplemented with 10% bovine calf serum until confluent, and the medium was exchanged every 2–3 days. To induce adipocyte differentiation, the 3T3-L1 cells were seeded in a 96-well plate. At confluency, differentiation was induced by changing the medium to MDI medium (DMEM containing IBMX, 111 µg/mL; DEX, 2 µM; and insulin, 2 µg/mL). For experiments, the cells were treated with bvPLA2 or PBS (vehicle) at the same time. The differentiation medium (MDI) was exchanged every 2 days. After 4 days of differentiation, fully differentiated cells were analyzed using oil red O staining. To culture bone marrow-derived macrophages (BMDMs), bone marrow was flushed and filtered through a 70-μm filter (BD Biosciences, Oxford, UK) from the femurs and tibiae of C57BL/6 mice. Bone marrow cells were cultured for 7 days in DMEM supplemented with 10% FBS, penicillin/streptomycin (100 U/mL, 100 μg/mL), and 20 ng/mL macrophage colony-stimulating factor (Sigma, St Louis, MO). To induce polarization, BMDMs were stimulated for 24-72 h with 100 ng/mL LPS for M1 activation, or 20 ng/mL IL-4 and IL-13 for M2 activation. Unstimulated BMDMs were indicated as M0. Coculture of adipocytes and BMDMs was performed in contact system. 3T3-L1 adipocytes were cultured and differentiated in 96-well plates. At day 4, BMDM macrophages were plated onto the differentiated 3T3-L1 adipocytes and cultured with PBS and bvPLA2 treatment for 24 h and then oil red O staining was performed.

### Oil red O staining

Differentiated 3T3-L1 cells were washed twice with PBS and fixed in buffered 4% paraformaldehyde for 20 min. Oil red O (Sigma-Aldrich St. Louis, MO, USA) was dissolved in 60% isopropanol, and the cells were stained with this oil red O solution for 20 min at RT (21–23 °C). After the cells were washed twice with distilled water, the OD at 510 nm was measured.

### Quantitative real-time PCR

Total RNA was isolated from WAT samples using the easy-BLUE RNA extraction kit (iNtRON Biotechnology, Korea) and cDNA was synthesized using Cyclescript reverse transcriptase (Bioneer, Korea). The synthesized cDNA was used for quantitative real-time PCR with the SensiFAST SYBR no-Rox kit (Bioline, Korea) (Cycling conditions: 95 °C for 15 s, 55 °C for 10 s and 72 °C 10 s). Each reaction was performed in triplicate. The base sequences of the primers were as follows: GAPDH: forward, 5′-CCC AGA AGA CTG TGG ATG G-3′; reverse, 5′-CAC ATT GGG GGT AGG AAC AC-3′. TNF-α: forward, 5′-TTC TGT CTA CTG AAC TTC GGG GTG ATC GGT CC-3′; reverse, 5′-GTA TGA GAT AGC AAA TCG GCT GAC GGT GTG GG-3′. IL-1β: forward, 5′-GGA CAG AAT ATC AAC CAA CAA GTG ATA-3′; reverse, 5′-GTG TGC CGT CTT TCA TTA CAC AG-3′. IL-12a: forward, 5′-GCT CTA GAC CCT GTG CCT TG-3′; reverse, 5′-GAA GGC TTA CCT GCA TCA GC-3′. IL-4: forward, 5′-ACG AAG AAC ACC ACA GAG-3′; reverse, 5′-TGA TGT GGA CTT GGA CTC-3′. CD206: forward, 5′-AGT GGC AGG TGG CTT ATG-3′; reverse, 5′-GGT TCA GGA GTT GTT GTG-3′. Ym1: forward, 5′-CAT TCA GTC AGT TAT CAG ATT CC-3′; reverse, 5′-AGT GAG TAG CAG CCT TGG-3′. PPAR γ: forward, 5′-GAA GGC TGA AGT CAC CAA GC-3′; reverse, 5′-TCA GCC TTG CCA GAG TTT TT-3′. C/EBP α: forward, 5′-TTA CAA CAG GCC AGG TTT CC-3′; reverse, 5′-CTC TGG GAT GGA TCG ATT GT-3′. UCP-1: forward, 5′-TCT CAG CCG GCT TAA TGA CT-3′; reverse, 5′-GCT GGG TGT ATG TGC CTT TT-3′.

### H&E and immunofluorescence staining

Liver, kidney, and eWAT tissues were fixed with 4% paraformaldehyde overnight. After dehydration and paraffin embedding, the tissues were sectioned (4 μm thick). The sections were deparaffinized with xylene and rehydrated using graded ethanol (100%, 90%, 80%, and 70%). The rehydrated tissue sections were washed under tap water and stained with hematoxylin solution for 5 min. After dipping in 1% acid alcohol, the sections were stained with eosin solution for 3 min. Then, the tissues were dehydrated with graded ethanol (70%, 80%, 90%, and 100%) and cleared with xylene for mounting. For immunofluorescence staining, eWAT tissues were immediately frozen in Tissue-Tek O.C.T Compound (Sakura Finetek USA, Torrance, CA). Ten-micrometer–thick cryosections were cut and placed on microscope slides. After fixation with 4% paraformaldehyde, the slides were washed with PBS for 15 min. The tissue was blocked with 1.5% BSA for 1 h and incubated overnight at 4 °C with rat anti-mouse CD11c and goat anti-rabbit CD163 (1:200; Serotec). After washing with PBS and the tissue was stained with goat anti-rabbit secondary Ab conjugated to Alexa Fluor 594 and rat anti-mouse Alexa Fluor 488 (1:500; Abcam, UK) for 2 h at RT. Slides were mounted and detected using laser scanning confocal microscopy (Carl Zeiss, Jena, Germany). Staining intensities of images were quantified with Image J software (National Institutes of Health, Bethesda, MD, USA).

### FACS staining and flow cytometry

WAT samples were digested with collagenase B (1 mg/mL; Roche), and DNaseI (1 Unit/mL; Roche) in serum-free DMEM (Welgene) medium for 20 min at 37 °C in a shaking incubator. Then, the tissue was dissociated using a gentle MACS Dissociator and a MACS C tube (Milteny Biotec). The isolated cells obtained were filtered through a 100-μm strainer (falcon) and centrifuged. After the red blood cells were removed by incubation in RBC lysis buffer, single cells were washed and stained with the following antibodies: CD45-FITC, CD11b-V510, CD206-APC, and CD11c-APC/cy7. All antibodies were purchased from e-bioscience (San Diego, CA, USA). Flow cytometry analysis was performed using a FACSLyric system (BD bioscience, CA, USA).

### Statistical analysis

The results are expressed as the mean and standard error. Statistical significance was analyzed by one-way ANOVA and the Newman–Keuls test using Prism 5 software. Two-way ANOVA was used for body weight analysis. All data are represented as the mean ± SEMs; *P < 0.05, **P < 0.01, ***P < 0.001 versus the ND group. ^#^*P* < 0.05, ^##^*P* < 0.01, ^###^*P* < 0.001 versus the HFD group.

## Results

### bvPLA2 reduces body weight and AT weight in HFD-induced obesity

**Fig. 1:**
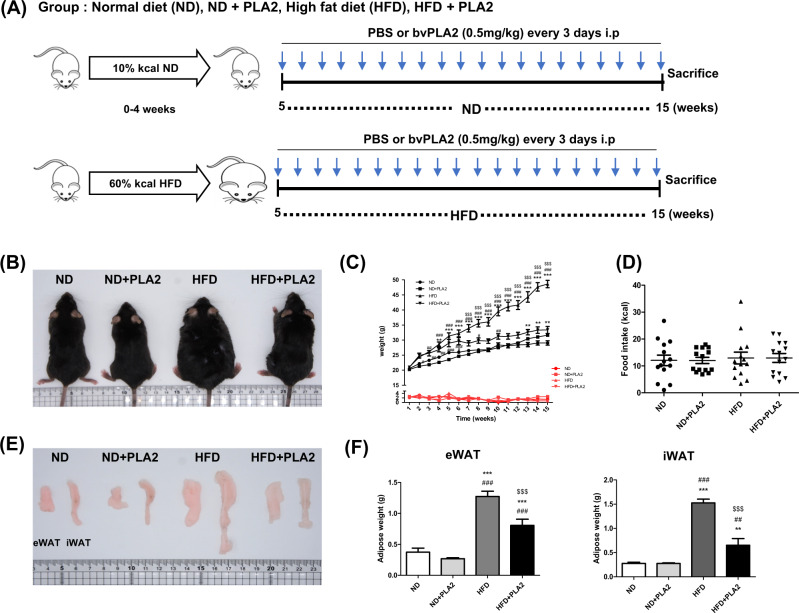
bvPLA2 inhibited HFD-induced obesity. **A** Experimental schedule. Five-week-old mice were divided into four groups: the normal diet (ND), ND plus bvPLA2 (ND + PLA), high-fat diet (HFD), and HFD plus bvPLA2 (HFD + PLA2) groups. The ND and HFD groups were treated with PBS vehicle only. **B** Representative pictures of the mice in the ND, ND + PLA2, HFD, and HFD + PLA2 groups after 15 weeks. Comparisons of **C** body weight (black) and weekly food intake (red) in the ND, ND + PLA2, HFD, and HFD + PLA2 groups. **D** Food intake (kcal) also compared among the groups. **E** Representative pictures of epididymal white adipose tissue (eWAT) and inguinal white adipose tissue (iWAT) and **F** the eWAT and iWAT weights of the ND, ND + PLA2, HFD, and HFD + PLA2 mice after 15 weeks. All data are represented as the mean ± SEMs; **P < 0.01, ***P < 0.0001 versus the ND group; ^##^*P* < 0.01, ^###^*P* < 0.0001 versus the ND + PLA2 group; ^$$$^*P* < 0.0001 versus the HFD group; *n* = 6 (color figure online).

To assess the therapeutic effect of bvPLA2 on obesity, C57BL/6 mice were fed a ND or HFD for 15 weeks. After 4 weeks of ND (10.3 kcal/day) or HFD (14.0 kcal/day) feeding, bvPLA2 or PBS vehicle injection was initiated on week 5 (Fig. 1A). During the experiment, the body weight of the ND- and HFD-fed mice with or without bvPLA2 (PLA2) were measured weekly. The body weight of the HFD group was higher than the ND group. However, the body weight of the HFD + PLA2 group was markedly decreased compared to the HFD group, indicating that PLA2 suppressed the body weight increase. The ND group and the ND + PLA2 group did not show any significant difference (Fig. 1B, C). Food intake did not differ among the groups, indicating that bvPLA2 did not affect appetite (Fig. 1D). The weights of the epididymal WAT (eWAT) and inguinal WAT (iWAT) were increased in the HFD group compared to the ND, whereas these weights were significantly decreased in the HFD + PLA2 group compared to the HFD. There was no significant difference in the ND + PLA2 group compared with the ND (Fig. 1E, F). These results suggest that bvPLA2 significantly reduced body weight and AT weight in obesity without affecting food intake.

### bvPLA2 decreases hepatotoxicity and nephrotoxicity in obese mice

**Fig. 2:**
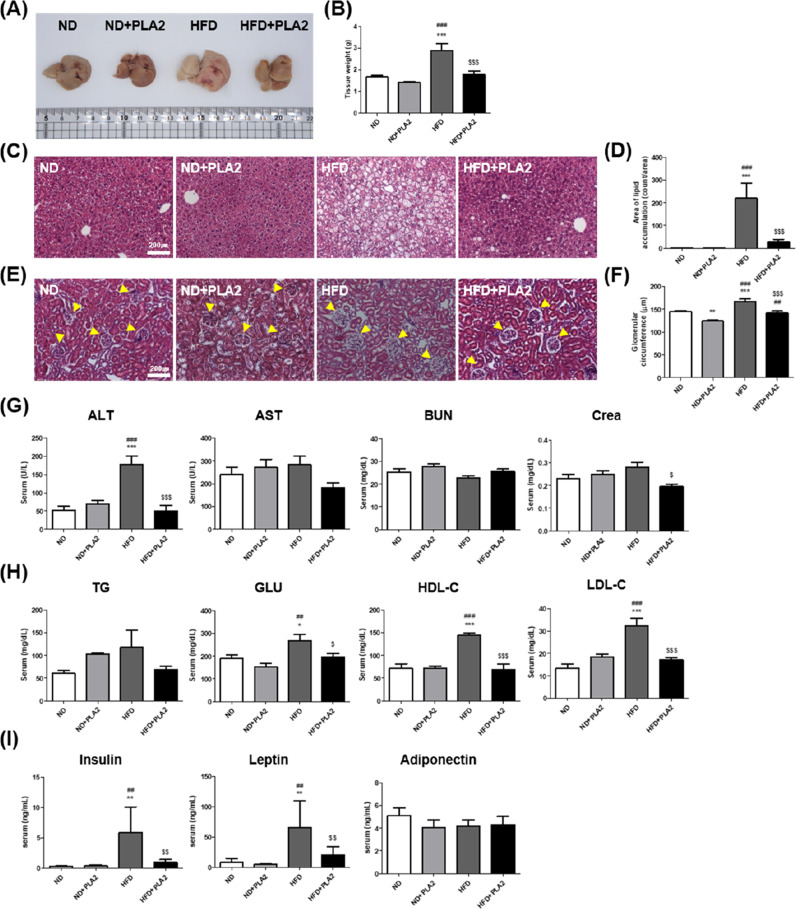
Histological analysis of liver and kidney tissue and metabolic regulation by bvPLA2 in HFD-induced obese mice. **A** Representative images of the liver and **B** measurements of liver weight. **C** Histology of H&E-stained liver and **D** area of lipid droplet accumulation in the liver. **E** Histology of H&E-stained kidney sections and **F** glomerular circumference expansion in the kidney. Analysis of serum levels of **G** ALT, AST, BUN, and Crea; **H** TG, GLU, HDL-C, and LDL-C; and **I** the hormones insulin, leptin, and adiponectin. All data are represented as the mean ± SEMs; *P < 0.05, **P < 0.01, ***P < 0.0001 versus the ND group; ^##^*P* < 0.01, ^###^*P* < 0.0001 versus the ND + PLA2 group; ^$^*P* < 0.05, ^$$^*P* < 0.01, ^$$$^*P* < 0.0001 versus the HFD group; *n* = 6.

To determine whether PLA2 attenuates HFD-induced hepatic steatosis and kidney inflammation, liver and kidney tissues from the mice were collected. Compared to the ND group, the size and weight of the liver in the HFD group were increased due to hepatomegaly, associated with lipid droplet accumulation. However, PLA2 treatment attenuated these increases in HFD-fed mice (Fig. 2A, B). Lipid droplet accumulation in the liver significantly increased in the HFD group compared to the ND group and decreased in the HFD + PLA2 group compared to the HFD group (Fig. 2C, D). Next, we examined the glomerular diameter as an early indicator of diabetic nephropathy and kidney damage. Hypertrophy, or increased glomerular diameter, in the kidneys due to lipid accumulation was observed in the HFD, and PLA2 was shown to alleviate the hypertrophy (Fig. 2E, F). These data indicate that PLA2 prevented hepatic steatosis and kidney damage by reducing lipid droplet accumulation.

To determine the effect of PLA2 on liver and kidney function, serum levels of alanine aminotransferase (ALT), aminotransferase (AST), blood urea nitrogen (BUN), and creatine (Crea) were measured. ALT level was significantly increased in the HFD. The increased levels of ALT and Crea in the HFD group were significantly reduced by PLA2 (Fig. 2G). TG, glucose (GLU), high-density lipoprotein cholesterol (HDL-C), low-density lipoprotein cholesterol (LDL-C), and hormone (insulin, leptin, and adiponectin) levels were analyzed to assess the effects of PLA2 on metabolic syndrome. The levels of GLU, HDL-C, and LDL-C were markedly higher in the HFD group, and these were significantly reduced in the HFD + PLA2 group compared to the HFD group. TG was also increased in the HFD group compared to the ND group, and slightly increased in the ND + PLA2 group compared with the ND group. This was decreased in the HFD + PLA2 group compared to the HFD group but without statistical significance (Fig. 2H). Plasma insulin and leptin levels were increased in the HFD group compared to the ND group and were significantly decreased in the HFD + PLA2 compared to the HFD group. However, adiponectin levels did not differ significantly among the groups (Fig. 2I). These results indicated that bvPLA2 alleviated HFD-induced metabolic dysfunction.

### bvPLA2 treatment prevents macrophage infiltration and regulates the M1 or M2-phenotypic markers as well as adipogenic factors in AT

**Fig. 3:**
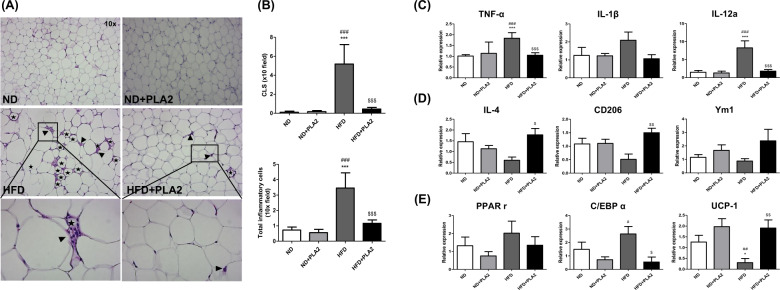
Treatment of bvPLA2 decreased the accumulation of macrophages in AT by controlling the M1 or M2-phenotypic markers and adipogenic factors. **A** Representative H&E-stained eWAT sections. The images in the black-lined box are magnified (bottom panel). Crown-like structures (CLS) formed by dead adipocytes are marked with stars, and infiltrated macrophages are indicated with black arrows head. **B** The number of CLS and infiltrated macrophages in adipose tissue. Relative expression levels of the **C** M1-like markers TNF-α, IL-1β, and IL-12a and **D** M2-like markers IL-4, CD206, and Ym1 in the adipose tissue were assessed by quantitative real-time PCR. The adipogenic markers **E** PPARγ, C/EBPα, and UCP-1 in the adipose tissue were analyzed by quantitative real-time PCR. All data represented as the means ± SEMs; *P < 0.05, ***P < 0.0001 versus the ND group; ^#^*P* < 0.05, ^##^*P* < 0.01, ^###^*P* < 0.0001 versus the ND + PLA2 group; ^$^*P* < 0.05, ^$$^*P* < 0.01, ^$$$^*P* < 0.0001 versus the HFD group; *n* = 6.

Given that obesity is characterized by the localization of crown-like structures (CLS), which are formed by pro-inflammatory macrophages surrounding dead adipocytes in AT [29], we next assessed whether bvPLA2 would reduce inflammatory macrophage infiltration in eWAT by histological examination of H&E-stained sections. ND mice showed few infiltrating inflammatory macrophages in eWAT. In comparison, HFD mice showed significantly increased CLS formation, whereas the HFD + PLA2 group showed dramatically decreased macrophage infiltration and CLS formation in AT (Fig. 3A, B).

The changes in macrophage phenotypes were determined by quantitative real-time PCR analysis. The expressions of TNF-α, IL-1β, and IL-12a were measured to verify the effect of bvPLA2 on M1-related markers. TNF-α and IL-12a expressions were significantly increased in the HFD group and decreased in the HFD + PLA2 group (Fig. 3C). In addition, the M2-related markers, IL-4 and CD206 were significantly increased in the HFD + PLA2 group compared to the HFD group (Fig. 3D). Next, the markers associated with lipid accumulation and adipogenesis such as PPARγ and C/EBPα were measured to investigate the effect of PLA2 on adipocytes; C/EBPα was significantly increased in the HFD and decreased by PLA2, but PPARγ showed no significant differences. On the other hand, UCP-1, the marker of thermogenesis, was significantly lowered in the HFD group, but significantly increased in the HFD + PLA2 group (Fig. 3E).

### bvPLA2 increases M2-like macrophage polarization and decreases M1-like macrophage polarization

**Fig. 4:**
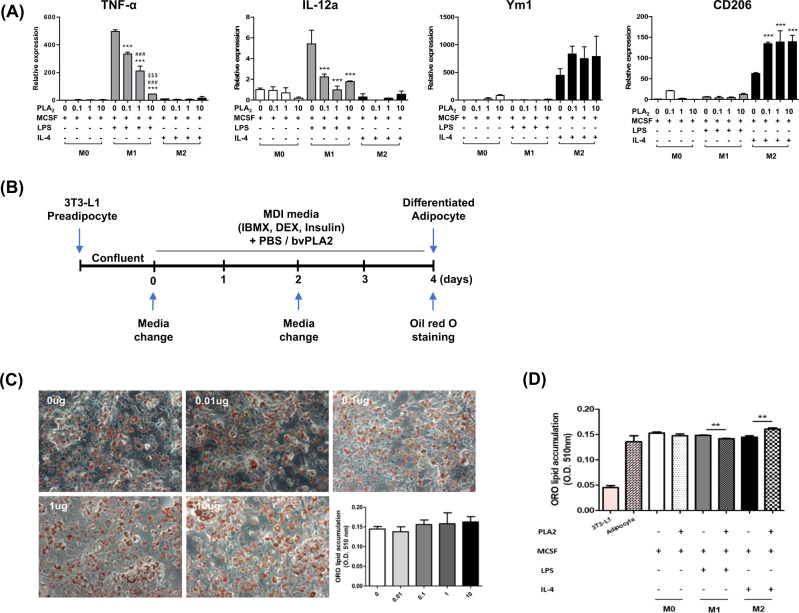
Treatment of bvPLA2 does not directly affect adipocytes but alters macrophage polarization. M1- and M2-differentiated BMDMs were treated with bvPLA2, and their respective marker gene expressions were evaluated by quantitative real-time PCR. mRNA expression levels of **A** M1-phenotypic markers TNF-α and IL-12a, and the M2-phenotypic marker Ym1 and CD206. Differentiated 3T3-L1 cells were treated with bvPLA2 (0–10 µg) (**B**) and stained with oil red O (**C**). Differentiated 3T3-L1 cells were cocultured with BMDMs during bvPLA2 treatment for 24 h and these cocultured cells were stained with oil red O (**D**). Data are normalized to the absorbance of the 0 µg group. All data are represented as the means ± SEMs; *P < 0.05, ***P < 0.0001 compared with the LPS only treated group; ^###^*P* < 0.0001 compared with the LPS 0.1 treated group; ^$$$^*P* < 0.0001 compared with the LPS-treated group; *n* = 3.

The effect of bvPLA2 on M1/M2 macrophage polarization was evaluated by measuring M1 and M2 differentiation markers in vitro. Bone marrow cells were differentiated into M0 macrophages by M-CSF supplementation. Then, the M0 macrophages were treated with LPS or IL-4 to stimulate polarization into M1 or M2 macrophages, respectively. LPS-treated M1 macrophages showed a significant increase in TNF-α and IL-12a mRNA expression compared to M0 macrophages, and these expressions were decreased by bvPLA2 treatment. Interestingly, IL-4-treated M2 macrophages increased Ym1 and CD206 mRNA expression, and this was further increased by PLA2. These results demonstrate that bvPLA2 inhibits M1 macrophage polarization and stimulates M2 macrophage polarization (Fig. 4A).

To clarify whether bvPLA2 directly reduced adipogenesis, pre-adipocytes (3T3-L1 cells) were differentiated into adipocytes in vitro and treated with bvPLA2 (Fig. 4B). No significant difference was observed between the control (0 µg) and bvPLA2-treated groups (0.01–10 µg) (Fig. 4C). These results suggest that bvPLA2 does not directly inhibit the differentiation of adipocytes but mediates macrophages. We also examined the effect of bvPLA2 in a coculture system of 3T3-L1 adipocytes and BMDMs. Cocultured cells were stained with oil red O. No significant difference was observed between the adipocytes and M0 with 10 µg of bvPLA2 treatment. Interestingly, adipocytes with M1 and M2 macrophages undergoing bvPLA2 treatment showed significantly decreased and increased lipid accumulations, respectively (Fig. 4D). From these results, it was confirmed that bvPLA2 directly regulates lipid accumulation through macrophages.

### bvPLA2 modulates M1/M2 macrophage ratio in AT

**Fig. 5:**
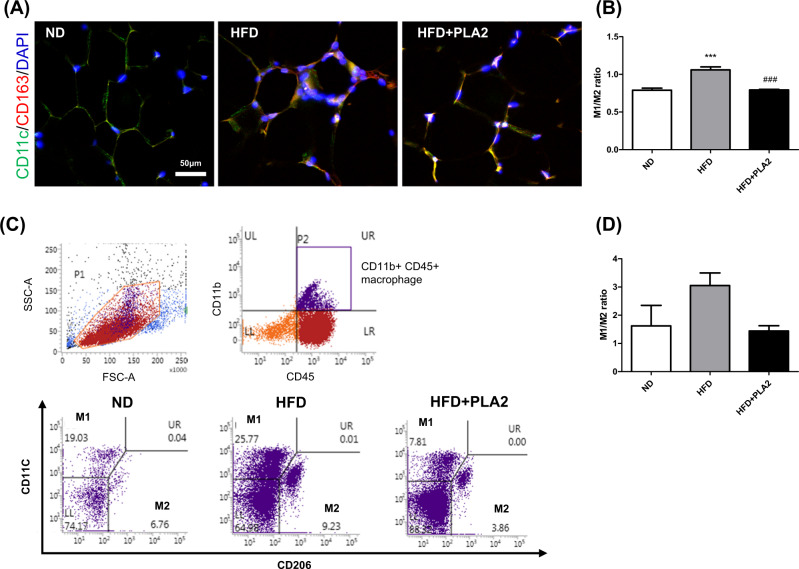
bvPLA2 treatment decreased the M1/M2 ratio in HFD-induced obese mice. **A**, **B** Frozen eWAT sections stained for CD11c (green), CD163 (red), and DAPI (blue) are shown. Scale bar in all images = 50 μm. **C** ATMs were stained with antibodies against CD45, CD11b, CD11c, and CD206 and examined by FACS analysis. Plots showing the percentages of M1/M2-like ATMs among the CD45^+^CD11b^+^ cells. **D** The ratio of classically activated M1-like ATMs (CD11c^+^CD206^−^) and alternatively activated M2-like ATMs (CD206^+^CD11c^−^) measured by FACS. All data are represented as the mean ± SEMs; ***P < 0.0001 versus the ND group; ^###^*P* < 0.0001 versus the HFD group; *n* = 4–5 (color figure online).

Immunofluorescence staining of CD11c and CD163 was performed to examine the changes in the M1/M2 macrophage ratio in AT. The M1/M2 ratio was significantly increased in the HFD compared to the ND group, whereas PLA2 significantly suppressed the M1/M2 ratio (Fig. 5A, B). Flow cytometry analysis also showed that the M1 (CD11c^+^CD206^−^)/M2 (CD11c^−^CD206^+^) macrophage ratio in the HFD group was increased over the ND group, and this imbalance was effectively reversed by bvPLA2 (Fig. 5C, D). These results suggest that bvPLA2 modulates the M1/M2 macrophage balances in AT.

### Anti-obesity effects of bvPLA2 are abolished in CD206-deficient mice

**Fig. 6:**
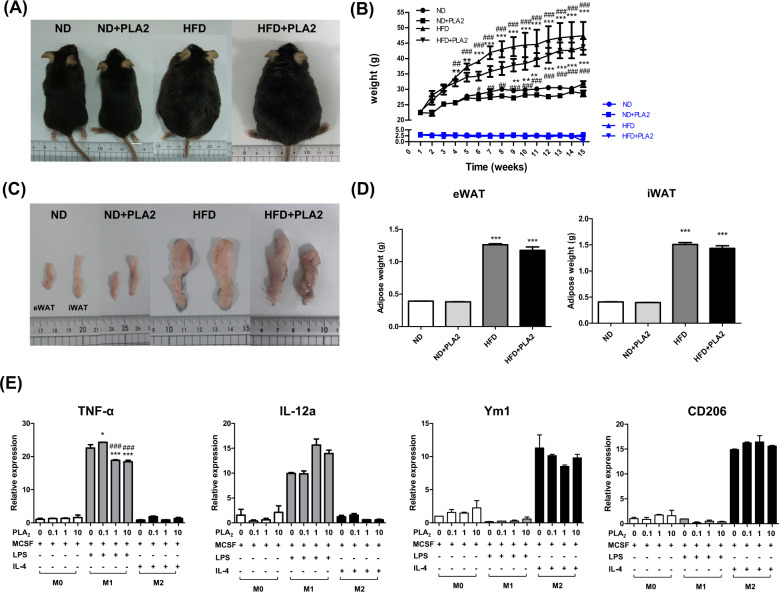
bvPLA2 inhibited HFD-induced obesity by mediating the mannose receptor CD206 but not Treg. **A** Representative pictures of CD206-deficient (CD206^−/−^) mice in the ND, ND + PLA2, HFD, and HFD + PLA2 groups after 15 weeks. **B** Comparisons of body weight (black) and weekly food intake (blue) in the ND, ND + PLA2, HFD. **C** Representative pictures of epididymal white adipose tissue (eWAT) and inguinal white adipose tissue (iWAT). **D** eWAT and iWAT weights of ND, ND + PLA2, HFD, and HFD + PLA2 mice after 15 weeks. **E** M1- and M2-differentiated CD206^−/−^ mouse bone marrow-derived macrophages were treated with bvPLA2, and their respective marker gene expressions were evaluated by quantitative real-time PCR. All data is represented as the mean ± SEMs; ***P < 0.0001 versus the ND group; ^###^*P* < 0.0001 versus the HFD group; *n* = 4 (color figure online).

In order to confirm the immunomodulatory effect of bvPLA2, CD206-deficient (CD206^−/−^) mice lacking the mannose receptor were used. The experiment was repeated in CD206^−/−^ mice with the same experimental schedule (Fig. 1A). The body weight and AT weight were measured and analyzed. There was no significant body weight change between the HFD and HFD + PLA2 groups (Fig. 6A, B). Furthermore, there was no significant AT weight differences between the groups either (Fig. 6C, D). M1/M2 macrophage polarization was also performed to evaluate the therapeutic effect of bvPLA2 with bone marrow cells from CD206^−/−^ mice (Fig. 6A). There were no significant changes in mRNA expression of Ym1 and CD206 (M2 macrophages marker) even with PLA2 treatment (Fig. 6E). However, M1 marker (TNF-α) was still decreased with PLA2 treatment in bone marrow cells from CD206^−/−^ mice. These results demonstrate that bvPLA2 specifically mediates mannose receptors (CD206) to modulate its anti-obesity effect.

To identify whether the therapeutic effect of PLA2 is related to regulatory T cells (Tregs) in HFD-induced obese mice, Treg depletion was performed by injecting anti-mouse CD25 antibody (clone PC61) every 3 days. Rat IgG was used as an isotype control. The body weight was significantly reduced in the IgG+PLA2 group compared with the IgG group in the obese mice. Similarly, body weight was markedly decreased in the PC61 + PLA2 group compared to the PC61-treated group. There was no significant difference between body weight in the IgG+PLA2 and PC61 + PLA2 groups (Supplementary Fig. 1), suggesting that the therapeutic effect of PLA2 is not mediated by Treg cells.

## Discussion

Obesity is a serious health problem both nationally and globally, and the current available treatments have variable efficacy or have a high risk of adverse effects. Currently, three drugs are approved by the US FDA for the treatment of obesity; orlistat, diethylpropion, and phentermine [30]. However, long-term treatment with these drugs is associated with several serious side effects, such as liver injury, kidney stones, and pancreatitis [31]. In addition, most anti-obesity drugs decrease body weight by inhibiting the absorption of fat from foods, resulting in steatorrhea [32]. Although regulating the inflammatory state is critical for the prognosis of obese patients, there is currently no available treatment for managing this aspect of obesity. Therefore, it is necessary to develop treatments that are more effective for obesity.

In this study, we demonstrated the beneficial effects on obesity of anti-inflammatory therapy using bvPLA2. It has been reported that bvPLA2 has anti-tumor, anti-inflammatory, and anti-obesity effects by reducing inflammation. The specific compound in bvPLA2 that contributes to its anti-obesity effect is not well established. To elucidate the therapeutic effects of bvPLA2 on inflammatory obesity, we used several HFD-induced obesity mouse models. Our results showed that bvPLA2 prevented rapid body weight gain by reducing adipocyte size in the WAT of the mice without affecting food intake (Fig. 1). This may explain that bvPLA2 contributed to the body weight effect without changing appetite, but energy expenditure or food absorption may different. Thus, further investigation will be required to confirm this issue.

AT, which is composed of adipocytes, is the main fat storage of the body as well as having metabolic control [33] and is divided into two types, white and brown AT (WAT and BAT, respectively) [34]. WAT contains large lipid droplets and releases adipokines (FFAs, TNF-α, IL-6, IL-10, resistin, leptin, and adiponectin) to maintain metabolic homeostasis and stores TG for future energy production during periods of malnutrition. In contrast, BAT contains multiple small lipid droplets and maintains body temperature and weight by converting consumed food into heat through UCP-1-mediated thermogenesis [35, 36]. Given that BAT can eliminate circulating GLU and TG during heat production, the browning of WAT, which means the switching of WAT to a BAT-like phenotype, might be a useful therapeutic strategy for obesity. To verify specific protective effects of bvPLA2 against the liver and kidney, we performed histological analysis in the HFD-induced mice. This showed that bvPLA2 prevented hepatic steatosis and glomerular hypertrophy in vivo. Importantly, it also prevented liver and kidney dysfunction and inhibited the elevation of GLU, cholesterol, and insulin levels in serum (Fig. 2). In addition, bvPLA2 significantly reduced obesity-induced macrophage infiltration into eWAT and subsequent CLS formation, which is related to adipocyte death. We also investigated whether bvPLA2 regulates M1 or M2 macrophages and adipogenic factors in obesity. M1/M2 lipid accumulation and adipogenesis-related markers were all examined (Fig. 3). M1 pro-inflammatory macrophages in the AT were significantly decreased, while M2 anti-inflammatory macrophages were increased by bvPLA2, suggesting that bvPLA2 stimulates M2-phenotypic macrophage differentiation. It has been shown that bvPLA2 significantly promotes UCP-1 and suppresses C/EBP α [30]. In our study, we suggested that bvPLA2 may participate in M1 and/or M2 polarization and thus control adipogenesis.

Several studies have reported that ATMs in lean individuals maintain homeostasis; however, the number of ATMs quickly increases in obese individuals. As obesity progresses, the accumulation of inflammatory M1 ATMs becomes a major source of pro-inflammatory cytokines such as TNF-α and IL-6, which can lead to insulin resistance via activation of endocrine signaling [16, 37]. Given that M2 ATMs maintain insulin sensitivity and homeostasis in AT [38], increased M2 polarization in ATMs is beneficial to improve insulin sensitivity. To clarify whether bvPLA2 directly reduced macrophage polarization or adipogenesis, BMDM and 3T3-L1 cells were used (Fig. 4). bvPLA2 stimulated M2 differentiation, while inhibiting M1 polarization but did not directly inhibit adipogenesis in vitro. In the present study, we also investigated the effect of bvPLA2 in an adipocyte and macrophage coculture system and observed the bvPLA2 enhances the anti-inflammatory response, indicating that it reduces lipid accumulation in the obese state. Moreover, we found that bvPLA2 modulated the M1/M2 macrophage ratio in AT (Fig. 5). In addition, it has been demonstrated that the characteristics of CD206^+^ ATMs exhibit selectively depleted CD206^+^ M2-like macrophages in CD206DTR mice [39]. They proved that CD206^+^ cells play an important role in WAT remodeling, and that CD206 can be used as a specific marker for M2-like ATMs. From these results, we demonstrated that bvPLA2 alleviates the M1/M2 imbalance in vivo.

We previously reported that bvPLA2 binds to the CD206 mannose receptor to promote Treg [25]. However, the specific mechanism by which bvPLA2 interacts with CD206 in macrophages, and its function, has not been confirmed. In this study, we tried to confirm the effect of bvPLA2 on macrophages. We used a CD206-deficient and Treg depletion mouse model to examine whether the therapeutic effect of bvPLA2 is associated with the mannose receptor and/or increased the Treg population in AT (Fig. 6). CD206^−/−^ mice showed no effective response to bvPLA2 treatment, indicating that bvPLA2 has immunomodulatory effects by binding to the CD206 receptor. Moreover, we found that depletion of Tregs by PC61 (anti-CD25 antibody) did not reverse the effect of bvPLA2, indicating that Tregs are not a major responder in bvPLA2 treatment in this model (Supplementary Fig. 1).

In conclusion, our study revealed a novel effect of bvPLA2 on macrophages. Here, we demonstrated that bvPLA2 has the therapeutic effect of reducing macrophage inflammation in the HFD-induced obesity mouse model. Thus, we propose that bvPLA2 has an important effect on the modulation of macrophage activity in a state of inflammation. In addition, we verified the potential of bvPLA2 as a therapeutic agent for obesity, through its modulation of the M1/M2 macrophages ratio.

## Supplementary information

Supplementary Figure

Merged File
