# Supplementary material for: Targeting of adipose tissue macrophages by bee venom phospholipase A2 attenuates high-fat diet-induced obesity
Source: Int J Obes (Lond). 2021 May 4;45(8):1656–67. doi: 10.1038/s41366-021-00823-4 (PMC8310798; doi:10.1038/s41366-021-00823-4)
Supplement: Supplementary file 1 — Supplementary Figure [file 41366_2021_823_MOESM1_ESM.pptx]

## Slide 1
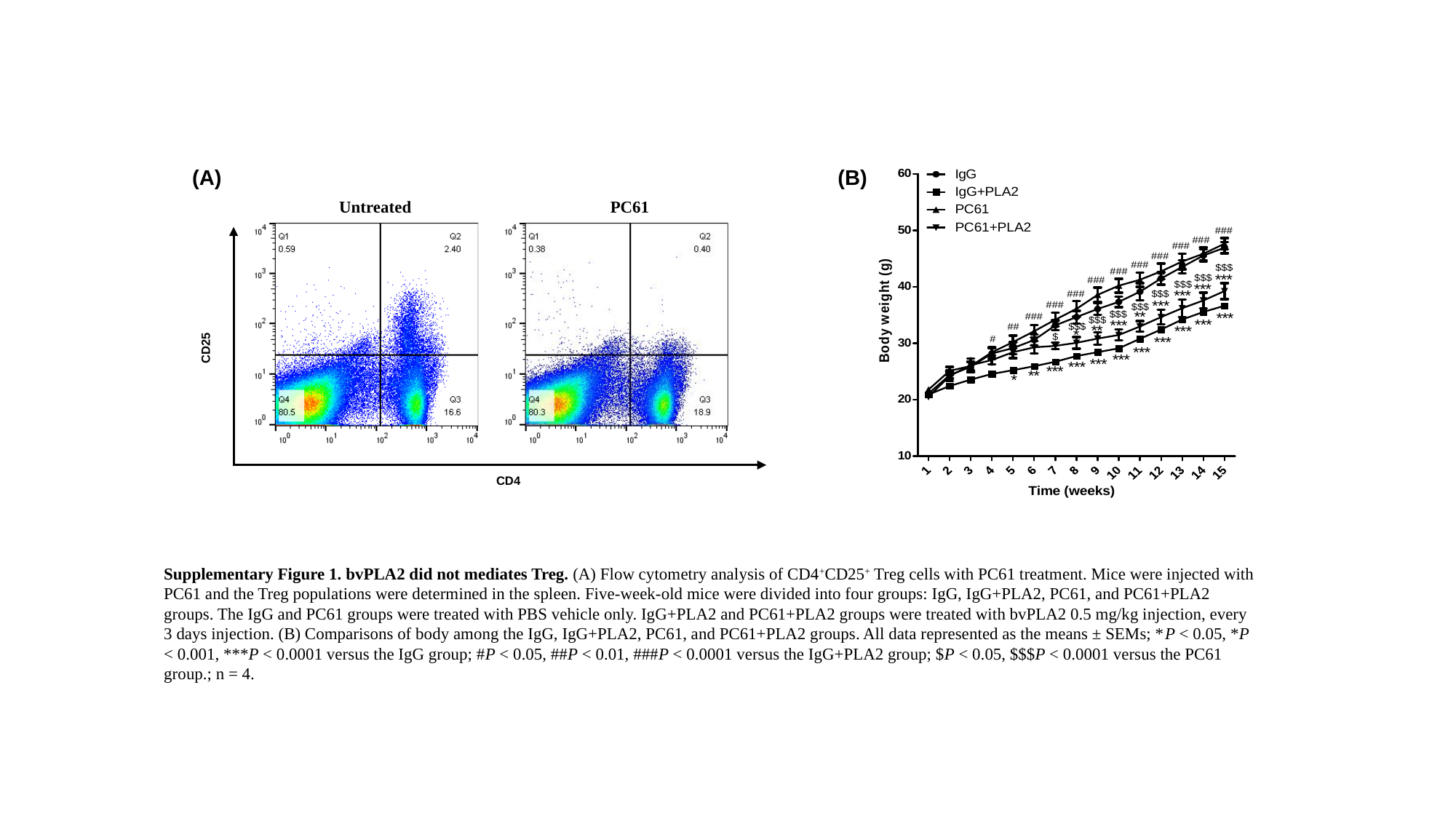

(A)
(B)
Untreated
PC61
CD25
CD4
Supplementary Figure 1. bvPLA2 did not mediates Treg. (A) Flow cytometry analysis of CD4+CD25+ Treg cells with PC61 treatment. Mice were injected with PC61 and the Treg populations were determined in the spleen. Five-week-old mice were divided into four groups: IgG, IgG+PLA2, PC61, and PC61+PLA2 groups. The IgG and PC61 groups were treated with PBS vehicle only. IgG+PLA2 and PC61+PLA2 groups were treated with bvPLA2 0.5 mg/kg injection, every 3 days injection. (B) Comparisons of body among the IgG, IgG+PLA2, PC61, and PC61+PLA2 groups. All data represented as the means ± SEMs; *P < 0.05, *P < 0.001, ***P < 0.0001 versus the IgG group; #P < 0.05, ##P < 0.01, ###P < 0.0001 versus the IgG+PLA2 group; $P < 0.05, $$$P < 0.0001 versus the PC61 group.; n = 4.
