# Supplementary material for: Targeting of adipose tissue macrophages by bee venom phospholipase A2 attenuates high-fat diet-induced obesity
Source: Int J Obes (Lond). 2021 May 4;45(8):1656–67. doi: 10.1038/s41366-021-00823-4 (PMC8310798; doi:10.1038/s41366-021-00823-4)
Supplement: Supplementary file 2 — Merged File [file 41366_2021_823_MOESM2_ESM.pptx]

## Slide 1
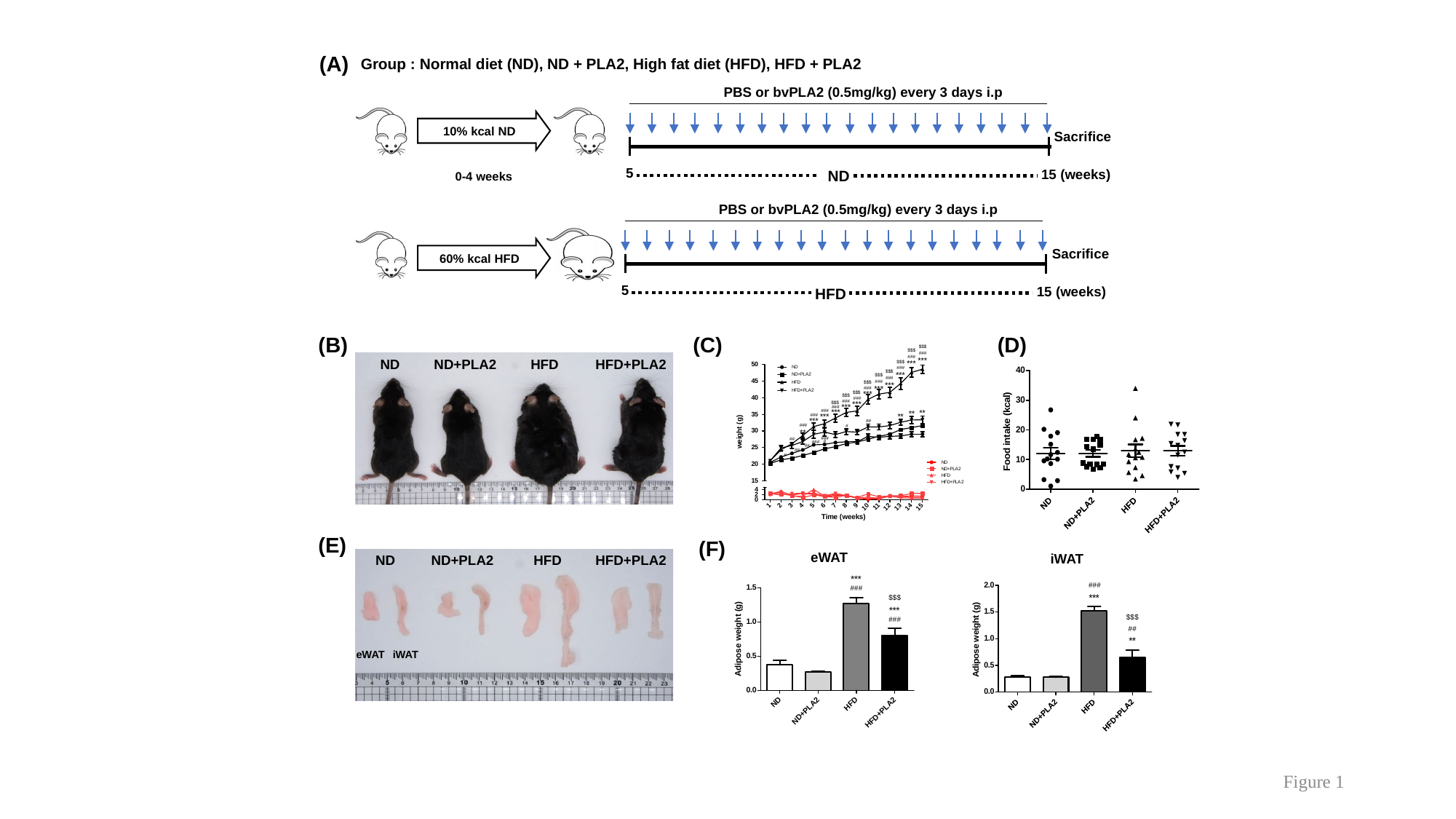

(A)
Group : Normal diet (ND), ND + PLA2, High fat diet (HFD), HFD + PLA2
PBS or bvPLA2 (0.5mg/kg) every 3 days i.p
Sacrifice
5
15 (weeks)
ND
10% kcal ND
0-4 weeks
PBS or bvPLA2 (0.5mg/kg) every 3 days i.p
Sacrifice
5
15 (weeks)
HFD
60% kcal HFD
(C)
(D)
(B)
ND
ND+PLA2
HFD
HFD+PLA2
(E)
(F)
eWAT
iWAT
ND
ND+PLA2
HFD
HFD+PLA2
eWAT
iWAT
Figure 1

## Slide 2
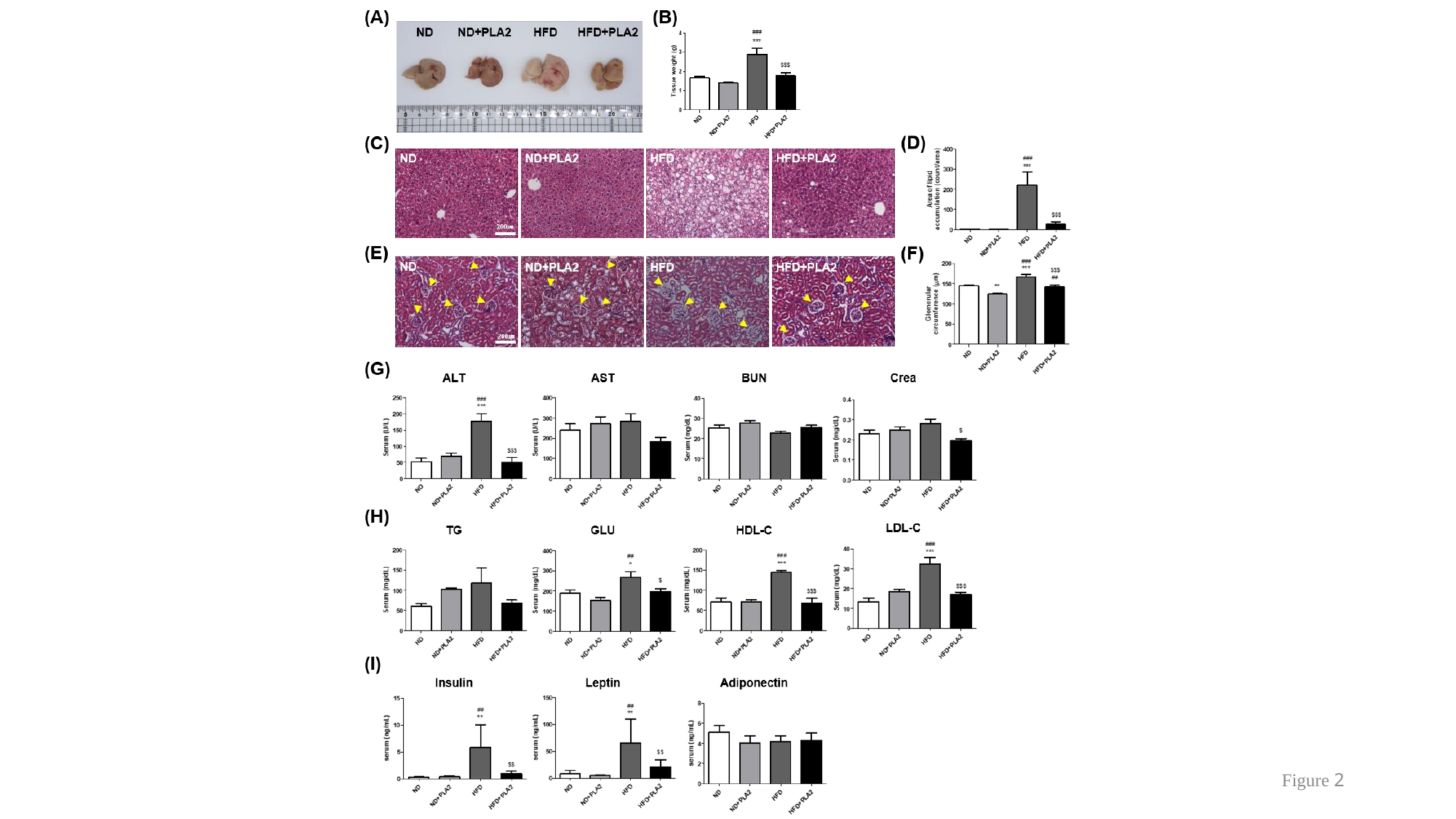

Figure 2

## Slide 3
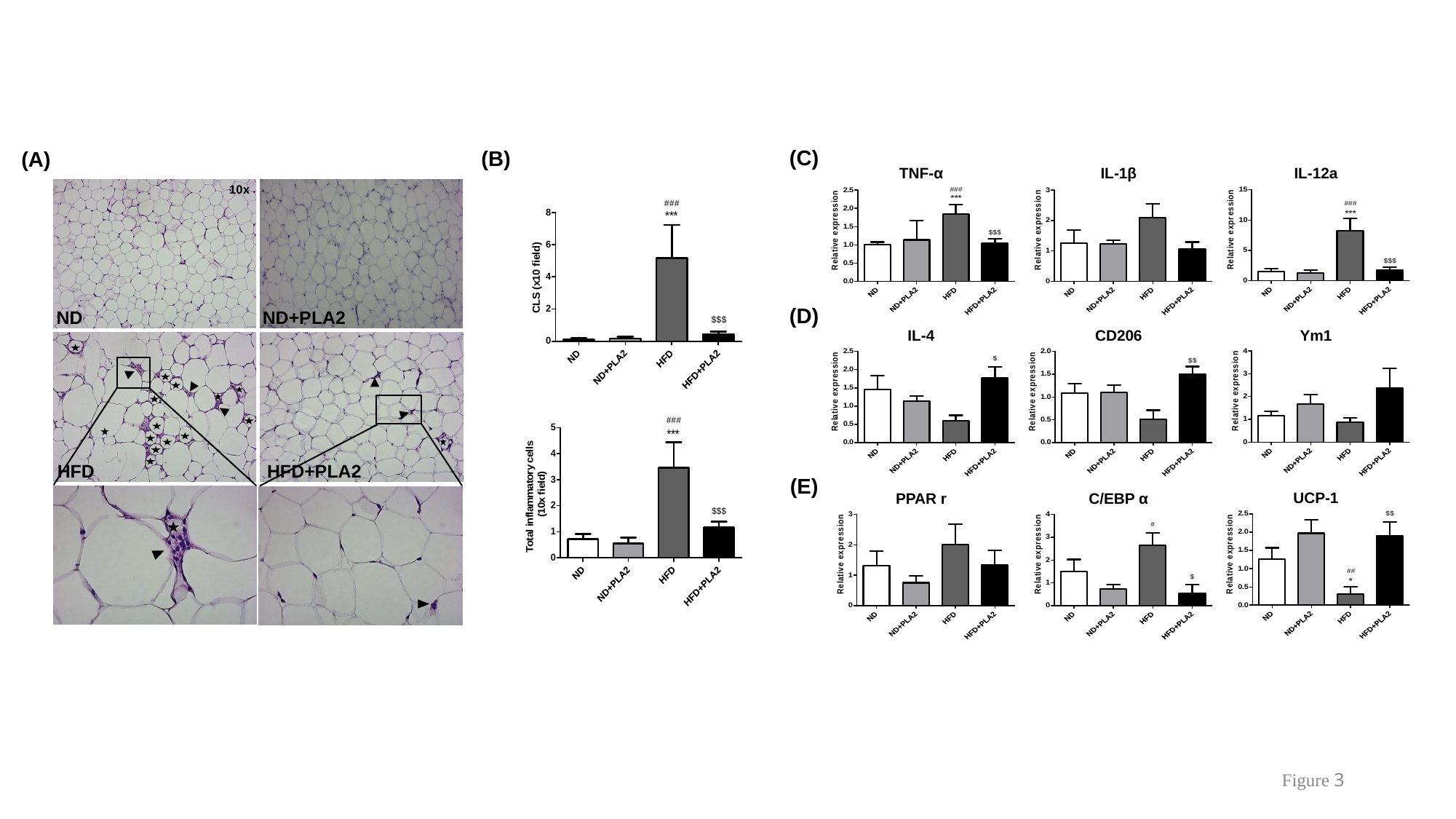

(C)
IL-12a
TNF-α
IL-1β
(D)
Ym1
IL-4
CD206
(E)
UCP-1
PPAR r
C/EBP α
(B)
(A)
10x
ND+PLA2
ND
10x
40x
HFD
HFD+PLA2
Figure 3

## Slide 4
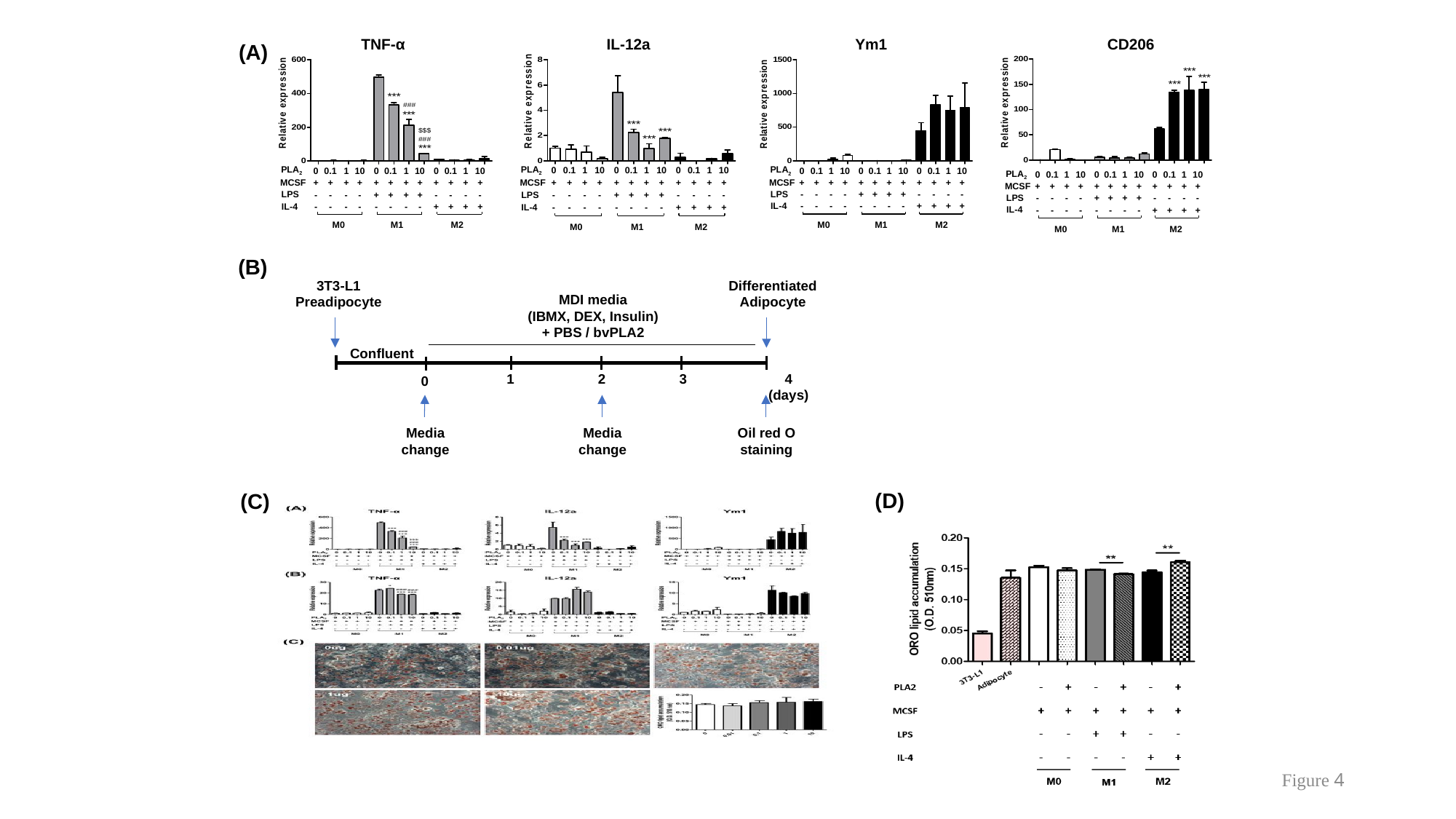

TNF-α
IL-12a
Ym1
CD206
PLA2
0
0.1
1
10
0
0.1
1
10
0
0.1
1
10
MCSF
+
+
+
+
+
+
+
+
+
+
+
+
LPS
-
-
-
-
+
+
+
+
-
-
-
-
IL-4
-
-
-
-
-
-
-
-
+
+
+
+
M0
M1
M2
PLA2
0
0.1
1
10
0
0.1
1
10
0.1
1
10
0
MCSF
+
+
+
+
+
+
+
+
+
+
+
+
LPS
-
-
-
-
+
+
+
+
-
-
-
-
IL-4
-
-
-
-
-
-
-
-
+
+
+
+
M0
M1
M2
PLA2
0
0.1
1
10
0
0.1
1
10
0
0.1
1
10
MCSF
+
+
+
+
+
+
+
+
+
+
+
+
LPS
-
-
-
-
+
+
+
+
-
-
-
-
IL-4
-
-
-
-
-
-
-
-
+
+
+
+
M0
M1
M2
PLA2
0
0.1
1
10
0
0.1
1
10
0
0.1
1
10
MCSF
+
+
+
+
+
+
+
+
+
+
+
+
LPS
-
-
-
-
+
+
+
+
-
-
-
-
IL-4
-
-
-
-
-
-
-
-
+
+
+
+
M0
M1
M2
(A)
(B)
3T3-L1 Preadipocyte
Differentiated
Adipocyte
MDI media
(IBMX, DEX, Insulin)
+ PBS / bvPLA2
Confluent
3
4 (days)
1
2
0
Media change
Media change
Oil red O staining
(D)
(C)
Figure 4

## Slide 5
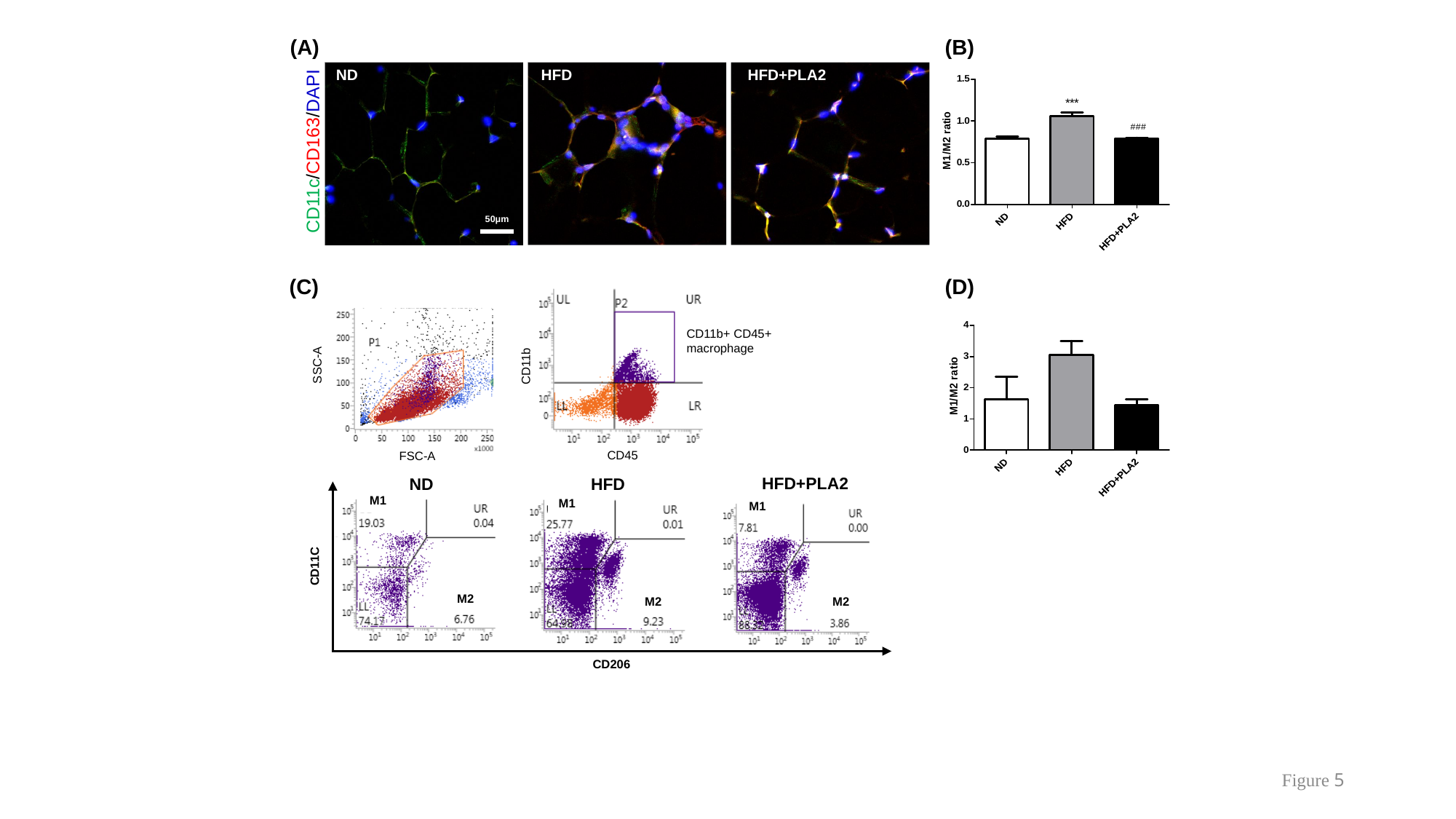

(A)
(B)
ND
HFD
HFD+PLA2
50μm
(C)
(D)
SSC-A
CD11b
CD45
FSC-A
HFD+PLA2
ND
HFD
CD11C
CD206
CD11c/CD163/DAPI
CD11b+ CD45+
macrophage
M1
M1
M1
M2
M2
M2
Figure 5

## Slide 6
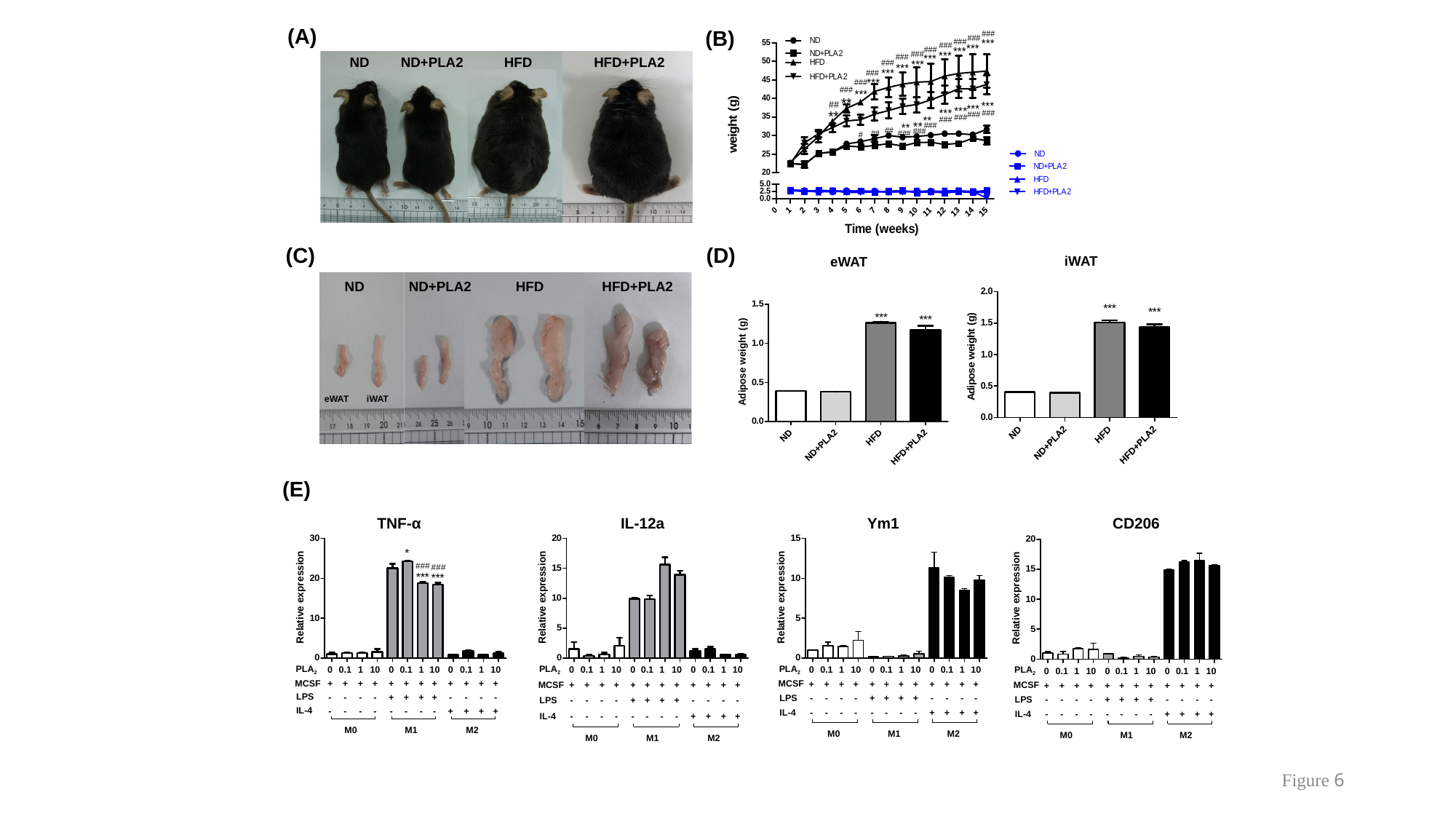

(A)
(B)
ND
ND+PLA2
HFD
HFD+PLA2
(C)
(D)
iWAT
eWAT
ND
ND+PLA2
HFD
HFD+PLA2
eWAT
iWAT
(E)
TNF-α
IL-12a
Ym1
CD206
PLA2
0
0.1
1
10
0
0.1
1
10
0
0.1
1
10
MCSF
+
+
+
+
+
+
+
+
+
+
+
+
LPS
-
-
-
-
+
+
+
+
-
-
-
-
IL-4
-
-
-
-
-
-
-
-
+
+
+
+
M0
M1
M2
PLA2
0
0.1
1
10
0
0.1
1
10
0
0.1
1
10
MCSF
+
+
+
+
+
+
+
+
+
+
+
+
LPS
-
-
-
-
+
+
+
+
-
-
-
-
IL-4
-
-
-
-
-
-
-
-
+
+
+
+
M0
M1
M2
PLA2
0
0.1
1
10
0
0.1
1
10
0
0.1
1
10
MCSF
+
+
+
+
+
+
+
+
+
+
+
+
LPS
-
-
-
-
+
+
+
+
-
-
-
-
IL-4
-
-
-
-
-
-
-
-
+
+
+
+
M0
M1
M2
PLA2
0
0.1
1
10
0
0.1
1
10
0
0.1
1
10
MCSF
+
+
+
+
+
+
+
+
+
+
+
+
LPS
-
-
-
-
+
+
+
+
-
-
-
-
IL-4
-
-
-
-
-
-
-
-
+
+
+
+
M0
M1
M2
Figure 6

## Slide 7
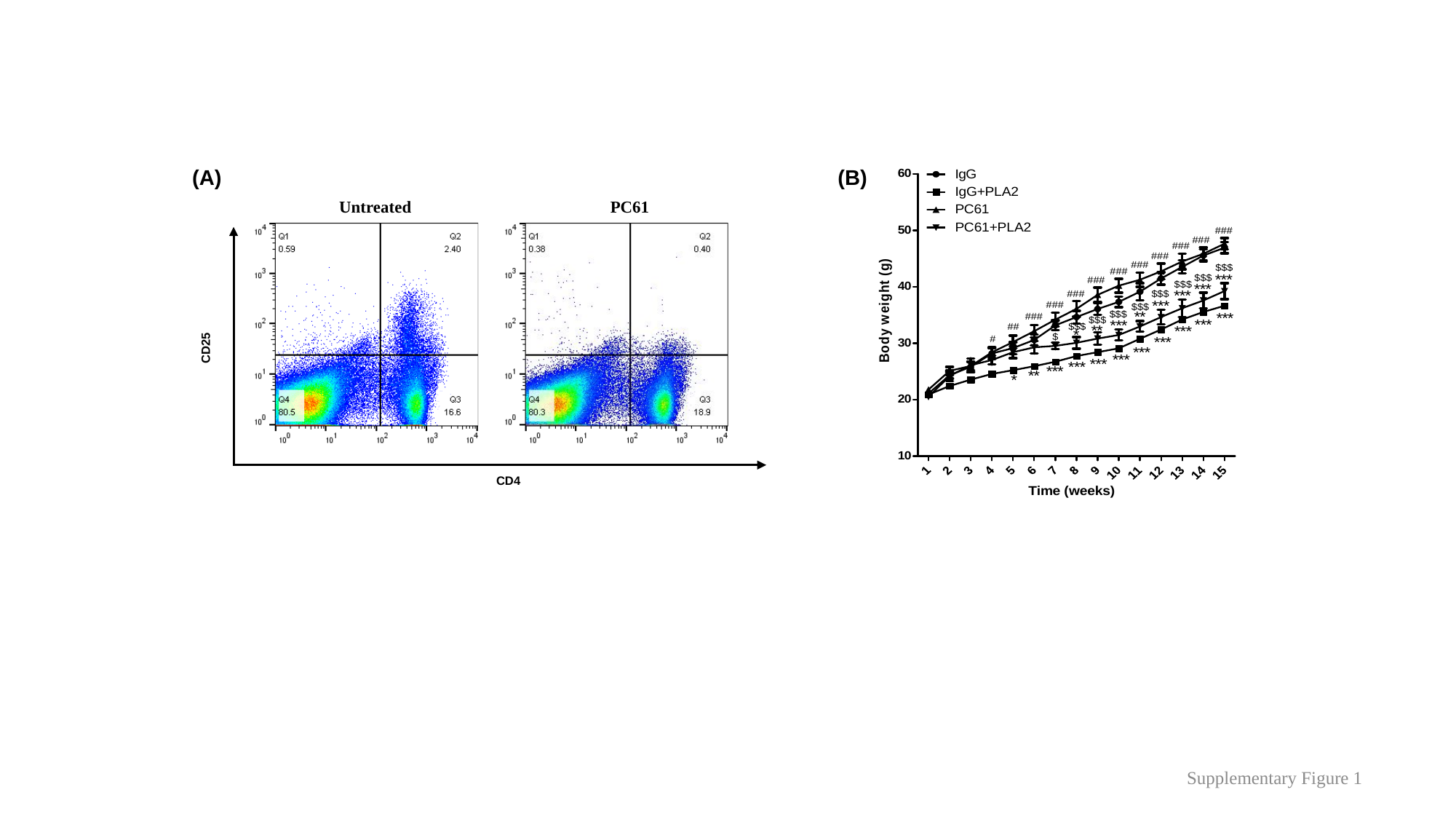

(A)
(B)
Untreated
PC61
CD25
CD4
Supplementary Figure 1
